# Impact of Adolescent and Young Adult Cancer Expertise in Oncologists on AYA Outcomes in Hodgkin Lymphoma: A Population‐Based Study in Ontario, Canada

**DOI:** 10.1002/cam4.71549

**Published:** 2026-01-30

**Authors:** Eden C. Andrew, Cindy Lau, Charlene Rae, Ronald D. Barr, Paul C. Nathan, Sumit Gupta

**Affiliations:** ^1^ Division of Hematology/Oncology Hospital for Sick Children Toronto Canada; ^2^ Children's Cancer Centre Royal Children's Hospital Melbourne Parkville Australia; ^3^ Cancer Research Program, ICES Toronto Canada; ^4^ Department of Pediatrics McMaster University Hamilton Canada; ^5^ Department of Pediatrics University of Toronto Toronto Canada; ^6^ Department of Paediatrics Faculty of Medicine, University of Toronto Toronto Canada

**Keywords:** adolescent and young adult, AYA, Hodgkin lymphoma, medical expertise

## Abstract

**Purpose:**

To determine whether adolescent and young adults (AYA) with Hodgkin lymphoma (HL) who are treated by oncologists with “AYA expertise” improve outcomes.

**Methods:**

All AYA aged 15–21 years diagnosed with HL in Ontario, Canada between 1992 and 2012 were identified, and clinical data abstracted as part of the IMPACT cohort. Linked administrative data were used to identify primary oncologists, defined as “AYA experts” if at diagnosis, ≥ 15% of the oncologist's previous 2 years of chemotherapy billings were for patients aged 15–29 years. Associations between seeing an AYA expert and outcomes were analysed.

**Results:**

Among 863 AYA with HL, 225 unique primary oncologists were identified. A total of 112 (13.0%) AYA had a primary oncologist with AYA expertise. Older patients [adjusted OR (aOR): 0.8 per year, 95% CI: 0.7–1.0; *p* = 0.04] and those seen in adult community hospitals [vs. regional cancer centre, aOR: 0.1, 95% CI: 0.02–0.4; *p* = 0.001] were less likely to see an AYA expert. Only 56 (6.4%) AYA received a fertility consult within 30 days of HL diagnosis; most occurred in the later study period (2006–2012). Seeing an AYA expert was associated with increased odds of fertility consultation (aOR: 2.1, 95% CI: 1.0–4.3; *p* = 0.04). Among the full cohort, there was no association between AYA expert care and event‐free survival (EFS), overall survival (OS), or subsequent live birth.

**Conclusion:**

A volume‐based definition of AYA expertise was associated with receipt of fertility consults, but not with EFS or OS for AYA with HL. If validated in other populations and settings, seeing a volume‐defined AYA expert could serve as a quality metric in AYA cancer care.

## 1 Background

A diagnosis of cancer during the challenging developmental transition from adolescence to adulthood can significantly disrupt life trajectories and impact physical and psychosocial wellbeing [1, 2]. In addition, adolescents and young adults (AYA) patients experience a unique distribution of cancer types compared with children or older adults; even within a single malignancy, tumour biology and treatment guidelines may still vary by age group [3, 4]. Consequently, AYA with cancer are a population with unique vulnerabilities and care needs at diagnosis, during and after cancer treatment [5]. It is recognized that traditional pediatric and adult models of care are suboptimal to cater for the specific needs of the AYA population; yet, the majority of patients will be treated in centers and by providers with limited AYA‐specific expertise [6].

Over the last 20 years, the field of AYA oncology has emerged as a recognized Area of Focused Competence and is a listed educational domain in medical oncology curricula [7, 8]. In order to provide best care for AYA, major international groups have called for investment in the development of specialized AYA oncology services staffed by clinicians with AYA‐specific expertise [9, 10, 11, 12]. Despite these recommendations, a general lack of AYA‐specific expertise among pediatric and adult medical oncologists persists. It is hypothesized that receiving care from an AYA expert will confer multiple benefits for AYA patients relating to their experience of healthcare, adherence to treatment, enrolment on clinical trials, psychosocial functioning, improved supportive care and survival [13]. Whether treatment by an AYA expert is associated with improved outcomes pertaining to survival or other measures of quality care, however, is unknown. More fundamentally, there is no consensus on how to define “AYA‐expertise,” apart from having undertaken specialized sub‐specialty fellowship training that is likely not feasible to disseminate widely [8].

In multiple other areas of medicine, higher volumes of a specific condition or patient population are associated with improved outcomes, either at an individual provider or institutional level [14, 15, 16]. Hodgkin lymphoma (HL) is the third most common cancer affecting Canadian AYAs aged 15–29 years with an age standardized incidence rate of 4.2 per 100,000 [17]; therefore this study will focus on this disease group. Also, HL treatment has historically carried a risk of infertility, a devastating potential long‐term effect of significant concern to AYA with cancer [18, 19]. Professional society guidelines have endorsed universal provision of fertility counselling to AYA at the time of cancer diagnosis, regardless of infertility risk [20]. Receipt of fertility counselling has been associated with improved decision‐making, improved coping, and diminished regret [21, 22].

The primary aim of this study was to determine whether a volume‐based definition of AYA expertise was associated with survival outcomes in a population‐based cohort of younger AYA (15–21 years of age at diagnosis) with HL. We also aimed to determine the association with referral for fertility consultation as an AYA‐specific quality of care metric, which may be generalizable to other aspects of AYA‐specific care.

## 2 Methods

### 2.1 Study Setting

In Canada, healthcare is separately administered by each province through universal insurance systems. Most physicians work on a fee‐for‐service basis, while those on alternative payment plans must submit shadow‐billing claims. The overall billing system in Ontario has remained the same for several decades. No organized AYA cancer care delivery system exists at a provincial level. Adolescents aged 15–18 years receive care at either pediatric or adult centers, while older AYA almost always receive care in adult centers [23]. Adult cancer care is available at both Regional Cancer Centers (RCCs) and community hospitals in Ontario.

### 2.2 IMPACT Cohort and Additional Data Sources

The Initiative to Maximize Progress in Adolescent and Young Adult Cancer (IMPACT) study collected patient, disease, treatment, and outcome data on all younger AYA aged 15–21 years diagnosed in Ontario between 1992 and 2012 with one of six malignancy types, including HL. Details have been published previously [24, 25]. Patients were identified through population‐based cancer registries; detailed demographic, disease, treatment, and outcome data were collected through chart abstraction and validated by clinicians. Patients were linked to population‐based health services databases housed at the ICES (formerly Institute for Clinical Evaluative Sciences), a research institute that holds an array of Ontario health‐related data (Tables S1–S5). ICES is an independent, nonprofit research institute whose legal status under Ontario's health information privacy law allows it to collect and analyze health care and demographic data, without consent, for health system evaluation and improvement. These datasets were linked using unique encoded identifiers and analyzed at ICES. These health services databases allowed the identification of hospitalizations, emergency room visits, physician encounters and longer term outcomes such as survival. Linkage to the ICES Physician Database (IPDB) allowed determination of physician characteristics. For these analyzes, we included only IMPACT Cohort patients with a first diagnosis of HL.

### 2.3 Outcomes

We selected both cancer‐related and quality of care‐related outcomes. The primary outcomes were event‐free (EFS) and overall survival (OS), both measured from the time of initial diagnosis. Events included relapse, progressive disease, death, and subsequent malignancy. Previously described and validated algorithms used health services data from chart abstraction and ICES datasets to identify late cancer events; these events were reviewed by investigators to ensure validity [26].

Receipt of a fertility consult within 30 days of initial cancer diagnosis was selected as our quality of care outcome [27]. Such consults were identified through physician visit billing with diagnostic codes for infertility [International Classification of Disease (ICD)‐9606 and 628, ICD‐10 N46.9 and N97.9] as validated in previous studies [27, 28]. Our final outcome, also related to fertility but restricted to female AYA, was live births as identified through MOMBABY, a population‐based database which links hospital admission records of delivering mothers and newborns [29, 30, 31].

### 2.4 AYA Expertise

**FIGURE 1:**
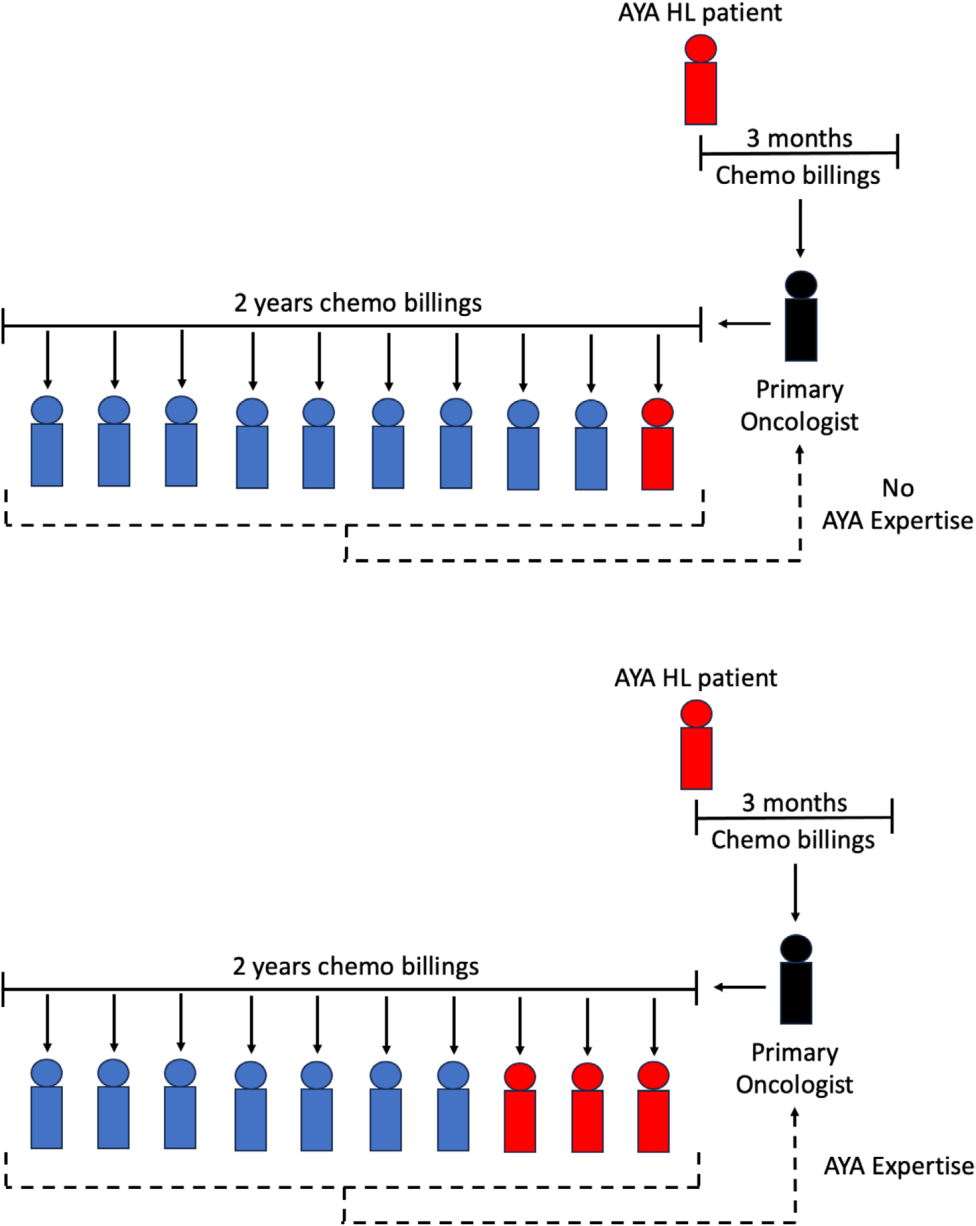
Definition and determination of AYA expertise.

We constructed a volume‐based definition of AYA expertise, our key predictor of interest. Each AYA's primary oncologist was identified, defined as the physician with the most chemotherapy billings for that specific patient within the first 3 months of the patient's HL diagnosis. The primary oncologist's chemotherapy billings for the prior 2 years were then identified from administrative databases, as were the unique patients represented by these billings (Figure 1). Primary oncologists were defined as having AYA expertise when ≥ 15% of this set of prior patients were between 15 and 29 years of age at the time of their cancer diagnosis. This age range was chosen as reflective of AYA practice in several Canadian jurisdictions [32, 33, 34]. Having AYA expertise was thus unique to each patient‐physician pair; a physician may have been considered as having AYA expertise for one patient but not for another. An alternative definition of ≥ 10% was also explored through sensitivity analysis.

### 2.5 Covariates

Patient‐level covariates included age at diagnosis and sex. Neighborhood income quintile and urban/rural status were determined using data from the Canadian census [35, 36]. Regional location was categorized as the 5 main Ontario health regions (Central, East, North, Toronto, and West). Locus of care (LOC) was categorized as pediatric versus adult centre based on the institution that delivered the majority of the first 3 months of therapy. Adult centers were further categorized as RCCs, as designated by Cancer Care Ontario, or community hospitals. Time period of diagnosis was defined as early (1992–1998), middle (1999–2005), or late (2006–2012). Physician characteristics included specialty, age at patient's cancer diagnosis, and the time since graduating from medical school at the time of the patient's cancer diagnosis. Disease‐level variables in the IMPACT cohort included histology and Ann Arbor stage. Patients were categorized as having either limited [Ann Arbor IA or IIA with no bulk (< 10 cm)] vs. advanced (all others) disease. Size of largest site of disease was also captured (≤ 5 cm vs. 6–9 cm vs. ≥ 10 cm). Treatment modality was categorized as chemotherapy vs. combined modality treatment.

### 2.6 Analyses

Patient, physician, disease, and treatment variables were summarized using descriptive statistics and where applicable compared across categories using chi squared tests or Fisher's exact tests for categorical variables as appropriate, and *t*‐tests for continuous variables. Predictors of seeing an “AYA expert” were determined using logistic regression models. Predictors of our primary outcomes were determined using either logistic regression for receiving a fertility consult, or Cox Proportional Hazard regression models for EFS and OS. Follow up end date for time to event analyses was December 31, 2016. The cumulative incidence of live births was compared between patients who were or were not cared for by an AYA expert using Grey's test. In all regression models, individual predictors significant at the *p* ≤ 0.1 level in univariate analyses were included in multivariable models even if the overall category for a group of covariates was not significant. Multicategorical variables were included if any category had a type 1 *p*‐value of < 0.1, regardless of the variable type 3 *p*‐value. As a key predictor of interest, AYA expert was retained regardless of univariate significance. In addition, in multivariable models of receipt of fertility consults, sex was retained regardless of univariate significance. We accounted for clustering of patients within individual physicians using generalized estimating equations. Sensitivity analyses using a 10% threshold definition of AYA expertise were conducted. Statistical significance was defined as *p* < 0.05. Analyses were performed using SAS, version 9.4 (SAS Institute, Cary, NC). Ethics approval was obtained at The Hospital for Sick Children and Sunnybrook Hospital. Informed consent was not required.

## 3 Results

Of 1014 AYA with HL, 863 (85.1%) had identifiable billings for chemotherapy within 3 months of their initial HL diagnosis. The majority of patients for whom no chemotherapy billings were identified were noted in IMPACT data to have received radiation or surgery as their primary treatment modality. Cohort characteristics are described in Table 1. The primary oncologists identified for these 863 AYA comprised 223 unique physicians. These 223 physicians had a median birth year of 1958 [interquartile range (IQR): 1951–1964] and a median year of graduation from medical school of 1984 [IQR: 1976–1990]. A total of 134 (60.1%) were male. Each unique physician was the primary oncologist for a median of three cohort AYA [IQR: 1–5].

**TABLE 1:** Characteristics of cohort patients (*N* = 863).

Age [years, median (IQR)]	18 (17–20)
Sex [*N* (%)]	
Male	422 (48.9)
Female	441 (51.1)
Time period [*N* (%)]	
Early (1992–1998)	250 (29.0)
Middle (1999–2005)	300 (34.8)
Late (2006–2012)	313 (36.3)
Locus of care [*N* (%)]	
Pediatric	232 (26.9)
Regional Cancer Center	426 (49.4)
Community Center	205 (23.8)
Neighborhood income quintile [*N* (%)]	
Rural	108 (12.5)
Urban Q1 (lowest)	128 (14.8)
Urban Q2	133 (15.4)
Urban Q3	149 (17.3)
Urban Q4	175 (20.3)
Urban Q5 (highest)	167 (19.4)
Region [*N* (%)]	
Central	268 (31.1)
East	225 (26.1)
North	49 (5.7)
Toronto	58 (6.7)
West	263 (30.5)
Stage [*N* (%)]	
I	55 (6.4)
II	505 (58.5)
III	149 (17.3)
IV	105 (12.2)
B symptoms [*N* (%)]	
No	467 (54.1)
Yes	346 (40.1)
Disease extent [*N* (%)]	
Limited	319 (37.0)
Advanced	495 (57.4)
Maximum deposit size [*N* (%)]	
≤ 5 cm	310 (35.9)
6–9 cm	167 (19.4)
> 10	122 (14.1)
Treatment modality [*N* (%)]	
Chemotherapy	393 (45.5)
Combined modality	455 (52.7)

Abbreviations: IQR, interquartile range; *N*, number.

**FIGURE 2:**
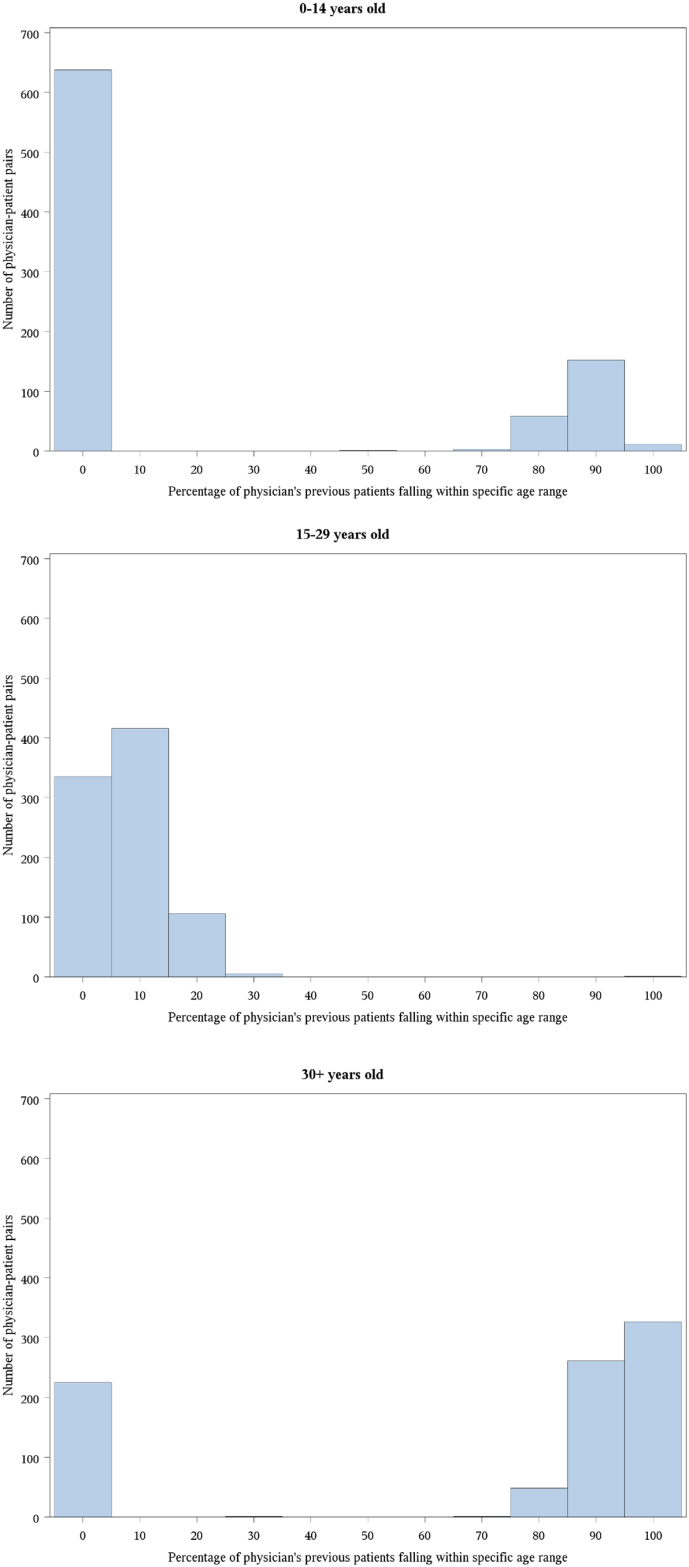
Proportion of primary oncologist's previous 2 years of patients falling within different age groups, across physician‐patient pairs (*N* = 863). For example, in Panel B, regarding the previously seen patient cohorts of each physician‐patient pair, 15–29 year olds made up 0%–5% of 338 of these cohorts (first bar), 5%–15% of 414 of these cohorts (second bar), and 15%–25% of 106 of these cohorts (third bar).

For each unique patient‐physician pair (*N* = 863), the median proportion of that physician's previous 2 years of patients (identified by chemotherapy billings) that were 15–29 year old was 7.6% (IQR: 2.8%–12.3%). The median proportion represented by those < 15 years old was 0.0% (IQR: 0.0%–80.6%) while that represented by those ≥ 30 year olds was 91.8% (IQR: 0.0%–97.1%). Distributions are illustrated in Figure 2.

Using a threshold of 15%, 112 (13.0%) patients had a primary oncologist meeting the definition of having AYA expertise. In multivariable analysis, older age and being seen in a community center were associated with lower odds of being seen by an AYA expert (Table 2). Of the 223 unique primary oncologists, 172 (77.1%) did not meet the definition of AYA expert, 16 (7.2%) did meet the definition, and 35 (15.7%) were considered an AYA expert for at least one cohort patient but not for another. AYA experts were both born and graduated later than AYA nonexperts but did not differ by sex (Table 3).

**TABLE 2:** Patient‐demographic variables associated with seeing an AYA expert.

	Univariate		Multivariable	
	OR (95% CI)	*p*	OR (95% CI)	*p*
Age at diagnosis (per year)	**0.7 (0.6–0.9)**	**< 0.001**	**0.8 (0.7–1.0)**	**0.04**
Sex				
Male	Ref	Ref	Ref	Ref
Female	1.0 (0.7–1.6)	0.88	1.1 (0.7–1.7)	0.78
Time period at diagnosis				
Early (1992–1998)	Ref	Ref	Ref	Ref
Middle (1999–2005)	2.1 (1.0–4.7)	0.06	0.6 (0.3–1.5)	0.28
Late (2006–2011)	0.8 (0.3–1.7)	0.47	1.9 (0.8–4.4)	0.15
Locus of care				
Pediatric	**3.0 (1.5–5.9)**	**0.001**	1.8 (0.8–3.9)	0.17
Regional Cancer Center	Ref	Ref	Ref	Ref
Community Center	**0.08 (0.02–0.3)**	**< 0.001**	**0.09 (0.02–0.4)**	**0.002**
Neighborhood income quintile				
Rural	0.9 (0.4–2.0)	0.86	—	—
Urban Q1 (lowest)	1.2 (0.7–2.0)	0.61	—	—
Urban Q2	0.9 (0.5–1.7)	0.79	—	—
Urban Q3	0.6 (0.3–1.4)	0.23	—	—
Urban Q4	1.0 (0.6–1.9)	0.89	—	—
Urban Q5 (highest)	Ref	Ref	Ref	Ref
Region				
Central	Ref	Ref	Ref	Ref
East	1.9 (0.8–4.1)	0.13	1.9 (0.8–4.2)	0.13
North	1.3 (0.4–4.1)	0.69	1.6 (0.6–4.9)	0.37
Toronto	1.8 (0.8–3.9)	0.15	2.3 (0.9–5.9)	0.09
West	**2.3 (1.0–5.3)**	**0.05**	**2.6 (1.1–6.2)**	**0.03**

*Note:* Bold indicates fields that reach statistical significance.

Abbreviations: CI, confidence interval; OR, odds ratio.

**TABLE 3:** Characteristics of AYA experts and non‐experts.

	AYA non‐expert (*N* = 172)	AYA expert (*N* = 16)	AYA expertise changed (*N* = 35)	*p*
Birth year (median, IQR)	1956 (1949–1963)	1973 (1953–1974)	1959 (1955–1966)	**0.02**
Sex (*N*, %)				
Male	107 (62.2)	5–9^a^	21 (60.0)	0.29
Female	46 (26.7)	5–9^a^	9–13^a^	
Medical school graduation year (median, IQR)	1984 (1975–1989)	1998 (1979–2000)	1985 (1982–1993)	**0.03**

*Note:* Bold indicates fields that reach statistical significance.

Abbreviations: AYA, adolescent and young adult; IQR, interquartile range; *N*, number.

^a^ Small cell sizes cannot be divulged due to privacy legislation.

Seeing an AYA expert was not associated with either EFS (HR: 1.1 [95% confidence interval (95% CI): 0.6–2.0], *p* = 0.81) or OS (HR: 1.3 [95% CI: 0.5–3.3], *p* = 0.63) (Tables S2 and S3). Variables associated with EFS on multivariable analysis included advanced disease extent (HR: 2.1 [95% CI; 1.1–3.9], *p* = 0.02) and treatment with chemotherapy alone (HR: 1.8 [95% CI: 1.2–2.6], *p* = 0.003) (Table 4). No disease‐related variables were associated with OS on multivariable analysis (Table 4).

**TABLE 4:** Variables associated with AYA outcomes.

	Association with event‐free survival (EFS)				Association with overall survival (OS)				Association with receipt of a fertility consult			
	Univariate		Multivariate		Univariate		Multivariate		Univariate		Multivariate	
	HR (95% CI)	*p*	HR (95% CI)	*p*	HR (95% CI)	*p*	HR (95% CI)	*p*	OR (95% CI)	*p*	OR (95% CI)	*p*
Age at diagnosis (per year)	1.0 (0.9–1.1)	0.59	—	—	1.0 (0.9–1.1)	0.9	—	—	0.9 (0.8–1.0)	0.11	—	—
Sex												
Male	Ref	Ref	Ref	Ref	Ref	Ref	Ref	Ref	Ref	Ref	Ref	Ref
Female	0.9 (0.7–1.2)	0.54	—	—	0.9 (0.6–1.3)	0.46	—	—	0.6 (0.3–1.0)	0.07	0.6 (0.3–1.2)	0.14
Time period at diagnosis												
Early (1992–1998)	Ref	Ref	Ref	Ref	Ref	Ref	Ref	Ref	Ref	Ref	Ref	Ref
Middle (1999–2005)	1.0 (0.7–1.4)	0.9	—	—	0.8 (0.5–1.4)	0.41	—	—	2.4 (0.9–6.7)	0.08	2.5 (0.9–6.6)	0.07
Late (2006–2011)	1.1 (0.7–1.7)	0.64	—	—	0.6 (0.3–1.2)	0.16	—	—	**4.8 (1.8–12.8)**	**0.002**	**4.8 (1.9–12.3)**	**0.001**
Locus of care												
Pediatric	1.2 (0.8–1.6)	0.42	—	—	1.1 (0.6–2.0)	0.78	—	—	1.8 (0.9–3.6)	0.11	—	—
Regional Cancer Center	Ref	Ref	Ref	Ref	Ref	Ref	Ref	Ref	Ref	Ref	Ref	Ref
Community Center	1.3 (0.9–1.8)	0.17	—	—	1.3 (0.7–2.3)	0.43	—	—	0.7 (0.3–1.6)	0.34	—	—
Neighborhood income quintile												
Rural	0.9 (0.5–1.4)	0.63	—	—	1.2 (0.6–2.2)	0.59	1.4 (0.6–3.2)	0.37	0.8 (0.3–2.1)	0.59	0.8 (0.3–2.2)	0.65
Urban Q1 (lowest)	1.2 (0.8–1.9)	0.36	—	—	1.0 (0.5–2.0)	0.96	0.9 (0.3–2.6)	0.91	**0.07 (0.01–0.5)**	**0.009**	**0.07 (0.01–0.5)**	**0.01**
Urban Q2	1.0 (0.6–1.5)	0.91	—	—	0.7 (0.3–1.5)	0.37	0.8 (0.3–2.0)	0.6	0.7 (0.3–1.6)	0.39	0.7 (0.3–1.8)	0.46
Urban Q3	0.7 (0.4–1.1)	0.14	—	—	**0.4 (0.2–1.0)**	**0.04**	0.5 (0.2–1.5)	0.22	0.7 (0.3–1.6)	0.39	0.7 (0.3–1.8)	0.49
Urban Q4	0.7 (0.4–1.1)	0.14	—	—	0.7 (0.3–1.4)	0.27	0.6 (0.2–1.5)	0.27	0.7 (0.3–1.7)	0.41	0.7 (0.3–1.7)	0.44
Urban Q5 (highest)	Ref	Ref	Ref	Ref	Ref	Ref	Ref	Ref	Ref	Ref	Ref	Ref
Region												
Central	Ref	Ref	Ref	Ref	Ref	Ref	Ref	Ref	Ref	Ref	Ref	Ref
East	1.1 (0.7–1.6)	0.65	—	—	1.0 (0.5–1.9)	0.95	0.8 (0.3–1.8)	0.55	**2.8 (1.3–6.3)**	**0.01**	**3.0 (1.3–6.7)**	**0.008**
North	1.1 (0.6–2.2)	0.71	—	—	**2.2 (1.0–4.9)**	**0.05**	**2.8 (1.1–7.2)**	**0.03**	0.7 (0.1–5.7)	0.72	0.8 (0.1–5.9)	0.78
Toronto	1.1 (0.6–2.1)	0.64	—	—	1.1 (0.4–3.0)	0.81	0.8 (0.2–3.1)	0.78	1.8 (0.4–8.4)	0.47	2.1 (0.4–11.0)	0.37
West	1.3 (0.9–1.9)	0.18	—	—	1.0 (0.5–1.9)	0.97	0.9 (0.4–2.0)	0.8	**3.6 (1.6–8.0)**	**0.002**	**3.8 (1.6–8.8)**	**0.002**
B symptoms^a^												
No	Ref	Ref	Ref	Ref	Ref	Ref	Ref	Ref	Ref	Ref	Ref	Ref
Yes	**1.8 (1.3–2.4)**	**< 0.001**	1.2 (0.8–1.8)	0.51	**2.4 (1.4–4.0)**	**< 0.001**	1.6 (0.7–3.3)	0.23	1.4 (0.8–2.7)	0.25	—	—
Disease extent												
Limited	Ref	Ref	Ref	Ref	Ref	Ref	Ref	Ref	Ref	Ref	Ref	Ref
Advanced	**2.5 (1.7–3.6)**	**< 0.001**	**2.1 (1.1–3.9)**	**0.02**	**4.0 (1.9–8.3)**	**< 0.001**	2.4 (0.9–6.7)	0.08	1.6 (0.8–3.1)	0.18	—	—
Maximum deposit size												
≤ 5 cm	Ref	Ref	Ref	Ref	Ref	Ref	Ref	Ref	Ref	Ref	Ref	Ref
6–9 cm	1.3 (0.9–2.1)	0.18	1.4 (0.9–2.2)	0.15	1.5 (0.8–3.2)	0.23	1.4 (0.6–2.9)	0.42	0.7 (0.3–1.8)	0.43	—	—
> 10	**1.6 (1.0–2.5)**	**0.04**	1.3 (0.8–2.2)	0.24	**2.5 (1.3–4.7)**	**0.006**	1.7 (0.9–3.3)	0.13	0.9 (0.4–2.4)	0.88	—	—
Treatment modality												
Chemotherapy	**1.5 (1.1–2.0)**	**0.007**	**1.8 (1.2–2.6)**	**0.003**	1.4 (0.8–2.3)	0.23	—	—	1.0 (0.6–1.7)	0.99	—	—
Combined modality	Ref	Ref	Ref	Ref					Ref	Ref	Ref	Ref
Seen by AYA expert	Ref	Ref	Ref	Ref								
No	1.4 (0.9–2.1)	0.15	1.1 (0.6–2.0)	0.81					Ref	Ref	Ref	Ref
Yes									**2.7 (1.4–5.1)**	**0.003**	**2.1 (1.1–4.0)**	**0.02**
Time between physician graduation and patient diagnosis (per year)									1.0 (0.96–1.04)	0.92	—	—

*Note:* Bold indicates fields that reach statistical significance.

Abbreviations: AYA, adolescents and young adults; CI, confidence interval; HR, hazard ratio.

^a^ Determined by chart abstraction defined as at least one of the following: Unexplained weight loss > 10% in the preceding 6 months; Unexplained recurrent fever ≥ 38°C in the preceding month; or Recurrent drenching sweats in the preceding month.

Of the 863 AYA, only 56 (6.5%) received a fertility consult as indicated through billing records within 30 days of their cancer diagnosis. This proportion varied throughout the study period. Only six of 250 (2.4%) AYA diagnosed during the early time period received a fertility consult vs. 17/300 (5.6%) of AYA diagnosed during the middle time period and 33/250 (13.2%) diagnosed in the late time period. Among the whole cohort, AYA who saw an AYA expert were more likely to receive a fertility consult than those who did not [adjusted odds ratio (aOR): 2.1, 95% CI: 1.1–4.0; *p* = 0.02], even when adjusting for time period, region, and neighborhood income quintile (Table 4). The same association was seen when restricting the cohort to those diagnosed in the middle or late time period (aOR: 2.4, 95% CI: 1.2–5.1; *p* = 0.02; Table S2) and when restricted to those diagnosed in the late time period (aOR: 2.7, 95% CI: 2.7, 95% CI: 1.1–6.5; *p* = 0.03; Table S3) with slightly larger magnitudes of association. Of note, in all multivariable models, AYA who lived in the poorest urban neighborhoods were substantially and statistically less likely to receive a fertility consult compared with those who lived in the richest urban neighborhoods. Among female AYA, no association was seen between having an AYA expert as their primary oncologist and subsequent liveborn birth (Table S5). Sensitivity analyses using a 10% threshold definition for AYA expertise did not yield substantively different results.

## 4 Discussion

In this study we demonstrate the utility of a volume‐based definition of AYA expertise, which after further validation could serve as a quality indicator in AYA cancer care. Over the study time period, fewer than one‐quarter of oncologists treating AYA with HL had cared for a patient cohort that included over 15% AYA patients in the preceding 2 years. Patients were more likely to see an AYA expert if they were younger and treated in a pediatric centre; however, only 13% of the total cohort of 863 AYA with HL were treated by an oncologist with AYA‐specific expertise. Recently, leading AYA oncology groups from Canada, Australia, Europe, and the US have called for the investment in training, recruitment and maintenance of a specialized AYA workforce, as a core component of quality care for AYA with cancer [8, 9, 10, 37]. Whether such calls have translated to more AYA patients being treated by healthcare providers with specialized expertise is unknown.

Interestingly, receiving care from an AYA‐expert did not translate to improved survival outcomes in this study. One explanation for why we did not observe a survival advantage through seeing an AYA expert in this study could be that HL is a disease for which excellent survival outcomes have been appreciated for over 30 years using a variety of treatment approaches [38], and therefore the potential to improve survival further using AYA‐specific expertise may be limited. It is possible that for AYA patients with other cancer types with poorer survival outcomes, such as sarcomas or high risk leukaemias, there may be a more appreciable survival benefit from seeing an AYA expert through their knowledge of both pediatric and adult approaches, and who may be more likely to facilitate clinical trial enrolment. Further, HL treatment protocols are typically outpatient‐based, less intensive and have less requirement to adhere to regular medications for prolonged periods of time when compared with other AYA cancers such as acute lymphoblastic leukemia or sarcomas, for whom seeing an AYA‐expert may offer more of a survival advantage through their skills in supporting adherence with recommended treatments and surveillance programs. Of note, although there have been historical reports of a survival advantage for patients with HL treated on pediatric protocols [39, 40], this association was not seen in our analysis of survival outcomes by treatment in pediatric versus adult settings. Our findings are consistent with a previous IMPACT cohort study conducted by our group [25], as well findings from another Canadian population‐based study from British Columbia [41], and a large retrospective analysis by the German Hodgkin Study Group who also found equivalent survival outcomes when patients were treated on pediatric or adult treatment protocols [42].

Oncologists with AYA expertise may be more likely to prioritize and attend to other key aspects of quality AYA care, such as mental and psychosocial health and oncofertility. In support of this hypothesis, we found that those who saw an AYA expert were over twice as likely to receive a fertility consultation as those who did not. Addressing oncofertility is a core component of holistic AYA care, and the awareness of the importance of oncofertility has risen in the last 20 years [43]. Our findings are consistent with this, with the proportion of AYA receiving a fertility consult increasing over fivefold from 2.4% to 13.2% for those diagnosed early (1992–1998) and late (2006–2012) respectively. Interestingly, despite the overall increase in fertility consults over time, the strength of association between seeing an AYA expert and receiving a fertility consult actually increased in magnitude over the study period. An important limitation in this study is that our definition (receipt of a fertility consult within 30 days of diagnosis as identified through billing codes) would not include those AYA who may have been counseled about fertility by the oncologist, but for whom referral for a specialized fertility consultation was either not indicated or declined by the AYA.

This study also highlights that overall, very few patients were referred for formal fertility counseling, despite clinical practice guideline recommendations that fertility risks are discussed and referral to fertility consultation expedited by treating oncologists [20, 44, 45]. Of particular concern is the association we found that AYA who lived in the poorest urban neighborhoods were less likely to receive a fertility consult compared with those who lived in the richest urban neighborhoods. Over the last decade with increased awareness of oncofertility, it is possible that fertility referral rates have continued to rise for patients seeing AYA or non‐AYA expert oncologists, and it would be interesting to apply our analysis to a more contemporary cohort. Consistent with our findings however, recent work has suggested that oncofertility referrals continue to occur inadequately, even at large academic institutions, which is in large part dependent on the practices of the treating oncologist [46, 47, 48]. We did not find an association between seeing an AYA expert and subsequent liveborn birth among the females in this cohort. However broader measures of infertility that take into account intention to conceive are required, but are difficult to measure using administrative data.

To our knowledge, this is the first study to investigate the impact of treatment by an expert in AYA oncology on patient outcomes. Previous studies within the broader oncology population are few and use a variety of techniques to measure expertise, but available data point toward the value of disease‐specific expertise. Most recently, a study of 874 Taiwanese patients with acute promyelomonocytic leukemia (APL) identified physician volume, defined as the total number of APL patients treated by each haematologist before treating the index patient, was independently associated with reduced mortality [49]. Another series of 1309 patients with chronic lymphocytic leukemia (CLL) treated at the Mayo clinic identified a clear association for shorter time to first treatment as well as improved OS for those patients who were seen by CLL‐experts compared with those who saw non‐CLL hematologists [50]. A UK population‐based study of 12,861 women with breast cancer whose survival outcomes were analyzed by caseload of each attending consultant, the majority of whom were surgeons [51] also utilized a volume‐based definition of expertise by estimating the median number of patients seen as primary consultations annually, calculated over the period from the first to last registration for that consultant. Patients who were treated by consultants with a case‐load of at least 30 patients per year had significantly improved survival outcomes compared with those who saw consultants with a case‐load of fewer than 10 patients per year (RR: 0.85 [0.77–0.93]).

Best quality of care for the AYA population probably requires a combination of both disease‐specific and age‐specific expertise. However, it is also likely that it is age and developmental‐specific expertise that most contributes to positive experiences of care. A survey of 176 AYA investigating their experience of cancer care clearly identified that their satisfaction with care was substantially higher if their healthcare team were sufficiently knowledgeable about AYA, irrespective of whether they were treated in an academic or community center [52]. Indeed, AYA‐specific expertise of the healthcare team was a more important determinant of patient satisfaction than any other aspect of care, including disease‐specific expertise.

Physician expertise is a complex, multifaceted concept that is challenging to both define and measure. It refers to the knowledge, skills, and attributes of people who perform at an exceptionally high level within a given domain [53]. Typically, medical experts have a specialised knowledge‐base, which was assessed through examinations and a certification process, and a skillset developed through subspecialty‐specific training and deliberate practice over an extended period of time [54]. Time‐related proxies for experience such as clinician age or years in practice have, somewhat counter‐intuitively, not been shown to predict expertise and may, in fact, be inversely associated with performance as measured by quality of care outcomes such as mortality [14, 55]. This was demonstrated in our study by the lack of association between years between physician graduation and patient diagnosis and receipt of fertility consultation (Table 4). Instead, direct measures of practice activity such as number or volume of specific patients cared for, as well as prior tests of medical knowledge, show a more consistent positive relationship with performance [14, 56, 57], which provides justification for the volume‐based definition of expertise employed in this investigation, although future work should aim to compare different measures of physician expertise (e.g., absolute vs. relative case volume) in order to determine the most valid metric in the oncology population. The oncologists who met the definition of expertise were born and graduated later than those who didn't, which may reflect the shift toward recognition of AYA as a unique population over time within the oncology fraternity.

This study has several strengths including its population‐based nature, the ability to identify individual oncologists and their previous patient populations, and the ability to identify both metrics of care quality (i.e., fertility consults) and long‐term outcome. Some limitations merit mention in addition to those discussed above, however. First, the selected cut‐off of at least 15% of the oncologist's previous 2 years of chemotherapy billings may not be the ideal definition of AYA expertise; while using a threshold of 10% did not result in substantively different findings, it is possible that other lookback windows and other thresholds may be more appropriate. There is also some potential for misclassification with identifying the primary oncologist through chemotherapy billings within 3 months of HL diagnosis, for example if primary oncologists had changed within that time period; in addition, non‐MD team members who would not generally submit billing claims but who may have met the definition of AYA expertise were not identifiable by billing data. Second, the studied treatment period between 1992 and 2012 is not representative of more recent trends in AYA cancer care, given that momentum within the field of AYA oncology has increased in the last 20 years, and with significantly more vigor in the last 10 years. However, the comprehensive nature of the data available for the IMPACT cohort justifies our selection of this patient population. Third, our results may lack generalizability to other healthcare systems, particularly to those without universal health insurance. Finally, our results pertain to a specific age subgroup (15–21 years at diagnosis), diagnosis (HL), and outcomes (EFS, OS, fertility consult). Future work should study the association of our proposed definition of AYA expertise with a variety of outcomes in multiple different AYA cancer populations and jurisdictions.

In conclusion, we found that AYA expertise as categorized using a volume‐based definition was associated with receipt of fertility consults but not with cancer survival among a population‐based cohort of younger AYA with HL. Further study into the impact of AYA expertise on survival outcomes is warranted to investigate the impact of AYA expertise in other disease groups. Given the curability of HL, it will be particularly important to evaluate the impact of AYA expertise on other outcomes reflective of quality AYA care in this population such as referral to psychosocial clinicians, adherence with treatment or enrolment on clinical trials. If validated in other populations and settings, seeing a volume‐defined AYA expert could serve as a quality metric in AYA cancer care.

## Author Contributions

**Eden C. Andrew:** writing – original draft (equal). **Cindy Lau:** data curation (lead), formal analysis (lead), writing – review and editing (supporting). **Charlene Rae:** writing – review and editing (equal). **Ronald D. Barr:** writing – review and editing (supporting). **Paul C. Nathan:** resources (equal), supervision (supporting), writing – review and editing (equal). **Sumit Gupta:** conceptualization (lead), formal analysis (equal), methodology (lead), resources (equal), supervision (lead), writing – original draft (equal).

## Conflicts of Interest

The authors declare no conflicts of interest.

## Supporting information


**Tables S1–S5:** cam471549‐sup‐0001‐TableS1‐S5.docx.

## Data Availability

Ontario privacy legislation prevents the disclosure of individual personal health information; study data thus cannot be made available.
