# Supplementary material for: Impact of Adolescent and Young Adult Cancer Expertise in Oncologists on AYA Outcomes in Hodgkin Lymphoma: A Population‐Based Study in Ontario, Canada
Source: Cancer Med. 2026 Jan 30;15(2):e71549. doi: 10.1002/cam4.71549 (PMC12856524; doi:10.1002/cam4.71549)
Supplement: Supplementary file 1 — Tables S1–S5: cam471549‐sup‐0001‐TableS1‐S5.docx. [file CAM4-15-e71549-s001.docx]

Supplemental Table 1. Population-based health services databases used to identify hospitalizations, emergency room visits, and physician encounters

| **Database** | **Data Elements** | **Description** | **Initiation Year** |
| --- | --- | --- | --- |
| ALR | Cancer therapies | Data elements pertaining to systemic cancer therapies, including radiation and chemotherapy | 2007 |
| DAD | Inpatient hospitalizations | One record per hospital admission including chart-abstracted demographic, clinical and outcome data. | 1988 |
| NACRS | ED visits | Demographic, clinical and disposition data. | 2000 |
| OHIP | Physician claims | Claims for services billed by fee-for-service Ontario physicians. Physicians under alternative funding plans are also required to submit shadow claims, ensuring capture of nearly all physician encounters. | 1991 |
| MOMBABY | Inpatient birth admission records | Links mothers and their newborns deterministically using inpatient birth admission records from DAD | 2002 |

ALR – Cancer Activity Level Reporting; DAD – Discharge Abstract Database; ED – emergency department; NACRS – National Ambulatory Care Reporting System; OHIP – Ontario Health Insurance Plan Claims Database

Supplemental Table 2. Variables associated with receipt of a fertility consult among AYA diagnosed in the middle and late time periods combined

|  | Univariate | | Multivariable | |
| --- | --- | --- | --- | --- |
|  | OR (95%CI) | p-value | OR (95%CI) | p-value |
| Age at diagnosis (per year) | 0.9 (0.7-1.0) | 0.12 | - | - |
| Sex |  |  |  |  |
| Male | ref | ref | Ref | ref |
| Female | 0.7 (0.4-1.4) | 0.35 | 1.0 (0.5-1.8) | 0.91 |
| Time period at diagnosis |  |  |  |  |
| Middle (1999-2005) | ref | ref | Ref | ref |
| Late (2006-2011) | 2.0 (1.0-4.0) | 0.06 | **2.1 (1.0-4.2)** | **0.04** |
| Locus of Care |  |  |  |  |
| Pediatric | 1.8 (0.9-3.8) | 0.11 | - | - |
| Regional Cancer Center | ref | Ref | Ref | ref |
| Community Center | 0.8 (0.3-2.1) | 0.70 | - | - |
| Neighborhood income quintile |  |  |  |  |
| Rural | 0.6 (0.2-1.9) | 0.40 | 0.8 (0.2-2.4) | 0.62 |
| Urban Q1 (lowest) | **0.08 (0.01-0.6)** | **0.01** | **0.08 (0.01-0.6)** | **0.01** |
| Urban Q2 | 0.8 (0.3-1.9) | 0.60 | 0.8 (0.3-2.3) | 0.73 |
| Urban Q3 | 0.6 (0.2-1.6) | 0.32 | 0.6 (0.2-1.6) | 0.29 |
| Urban Q4 | 0.8 (0.3-1.8) | 0.52 | 0.7 (0.3-1.8) | 0.43 |
| Urban Q5 (highest) | ref | ref | Ref | ref |
| Region |  |  |  |  |
| Central | ref | ref | Ref | ref |
| East | **2.4 (1.1-5.5)** | **0.03** | **2.6 (1.1-6.1)** | **0.03** |
| North | 0.8 (0.09-6.4) | 0.80 | 0.9 (0.1-6.7) | 0.89 |
| Toronto | 1.6 (0.4-7.8) | 0.53 | 2.0 (0.4-10.3) | 0.40 |
| West | **3.5 (1.5-7.8)** | **0.003** | **3.2 (1.3-7.5)** | **0.01** |
| B symptoms |  |  |  |  |
| No | ref | ref | Ref | ref |
| Yes | 1.4 (0.7-2.6) | 0.35 | - | - |
| Disease extent |  |  |  |  |
| Limited | ref | ref | Ref | ref |
| Advanced | 1.8 (0.9-3.7) | 0.10 | 1.6 (0.8-3.3) | 0.21 |
| Maximum deposit size |  |  |  |  |
| ≤5 centimeters | ref | ref | Ref | ref |
| 6-9 centimeters | 0.6 (0.2-1.6) | 0.28 | - | - |
| >10 centimeters | 1.1 (0.4-2.7) | 0.93 | - | - |
| Treatment modality |  |  |  |  |
| Chemotherapy | 1.0 (0.6-1.8) | 0.94 | - | - |
| Combined modality | ref | ref | Ref | ref |
| Seen by AYA expert |  |  |  |  |
| No | ref | ref | Ref | ref |
| Yes | **3.0 (1.5-5.8)** | **0.001** | **2.4 (1.2-5.1)** | **0.02** |

AYA – Adolescents and young adults; CI – confidence interval; OR - Odds ratio

Supplemental Table 3. Variables associated with receipt of a fertility consult among AYA diagnosed in the late time period

|  | Univariate | | Multivariable | |
| --- | --- | --- | --- | --- |
|  | OR (95%CI) | p-value | OR (95%CI) | p-value |
| Age at diagnosis (per year) | 0.9 (0.7-1.1) | 0.13 | - | - |
| Sex |  |  |  |  |
| Male | ref | ref | Ref | ref |
| Female | 1.1 (0.5-2.4) | 0.79 | 1.2 (0.5-2.7) | 0.69 |
| Locus of Care |  |  |  |  |
| Pediatric | 1.4 (0.6-3.7) | 0.45 | - | - |
| Regional Cancer Center | Ref | Ref | Ref | ref |
| Community Center | 1.0 (0.3-3.1) | 0.93 | - | - |
| Neighborhood income quintile |  |  |  |  |
| Rural | 0.6 (0.1-2.9) | 0.55 | 0.6 (0.1-3.1) | 0.54 |
| Urban Q1 (lowest) | **0.1 (0.02-0.8)** | **0.03** | **0.1 (0.01-0.7)** | **0.02** |
| Urban Q2 | 0.6 (0.2-2.1) | 0.45 | 0.6 (0.2-2.5) | 0.51 |
| Urban Q3 | 0.5 (0.2-1.6) | 0.25 | 0.6 (0.2-1.8) | 0.32 |
| Urban Q4 | 0.7 (0.2-2.3) | 0.57 | 0.7 (0.2-2.2) | 0.52 |
| Urban Q5 (highest) | ref | ref | Ref | ref |
| Region |  |  |  |  |
| Central | ref | ref | Ref | ref |
| East | 1.8 (0.7-4.8) | 0.25 | 1.9 (0.7-4.9) | 0.20 |
| North | 0.9 (0.1-8.1) | 0.93 | 1.0 (0.1-8.6) | 0.99 |
| Toronto | 2.3 (0.4-11.9) | 0.32 | 2.7 (0.5-15.8) | 0.27 |
| West | **2.7 (1.0-7.3)** | **0.05^a^** | 2.4 (0.8-6.9) | 0.11 |
| B symptoms |  |  |  |  |
| No | ref | ref | Ref | ref |
| Yes | 0.9 (0.4-1.9) | 0.74 | - | - |
| Disease extent |  |  |  |  |
| Limited | ref | ref | Ref | ref |
| Advanced | 1.5 (0.6-3.6) | 0.36 | - | - |
| Maximum deposit size |  |  |  |  |
| ≤5 centimeters | ref | ref | Ref | ref |
| 6-9 centimeters | 0.5 (0.2-1.9) | 0.32 | - | - |
| >10 centimeters | 0.7 (0.2-2.3) | 0.55 | - | - |
| Treatment modality |  |  |  |  |
| Chemotherapy | 1.4 (0.7-2.7) | 0.41 | - | - |
| Combined modality | ref | ref | Ref | ref |
| Seen by AYA expert |  |  |  |  |
| No | ref | ref | Ref | ref |
| Yes | **2.6 (1.1-6.2)** | **0.03** | **2.7 (1.1-6.5)** | **0.03** |

AYA – Adolescents and young adults; CI – confidence interval; OR – Odds ratio

^a^ p = 0.0486

Supplemental Table 4. Variables associated with receipt of a fertility consult among AYA diagnosed in the middle time period

|  | **Univariate^a^** | |
| --- | --- | --- |
|  | **OR (95%CI)** | **p-value** |
| Age at diagnosis (per year) | 0.9 (0.6 -1.3) | 0.6035 |
| Female | 0.3 (0.1-1.0) | 0.0541 |
| Male | ref |  |
| Locus of Care |  |  |
| Community Center | 0.9 (0.2 -3.6) | 0.8409 |
| Pediatric | 2.6 (0.7 -8.9) | 0.1362 |
| Regional Cancer Center | ref |  |
| Neighborhood income quintile |  |  |
| Rural | 0.8 (0.1-4.1) | 0.7481 |
| Urban Q1 & Q2 | 0.6 (0.2 -2.0) | 0.4041 |
| Urban Q3 | 0.9 (0.10- 5.2) | 0.8734 |
| Urban Q4 | 0.9 (0.2- 3.6) | 0.8639 |
| Urban Q5 (highest) | Ref |  |
| Region |  |  |
| Central | NA |  |
| East | NA |  |
| North | NA |  |
| Toronto | NA |  |
| West | NA |  |
| B symptoms |  |  |
| No | ref |  |
| Yes | 2.6 (0.9-7.7) | 0.0918 |
| Disease extent |  |  |
| Advanced | 2.3 (0.7 -7.4) | 0.1672 |
| Limited | ref |  |
| Maximum deposit size |  |  |
| ≤5 centimeters | ref |  |
| 6-9 centimeters | 0.6 (0.1 -6.0) | 0.6780 |
| >10 centimeters | 2.7 (0.5 -13.7) | 0.2393 |
| Treatment modality |  |  |
| Chemotherapy | 0.5 (0.2 , 1.6) | 0.2702 |
| Combined modality | ref |  |
| Seen by AYA expert |  |  |
| No | ref |  |
| Yes | 2.7 (0.9 - 8.1) | 0.0807 |

^a^ There were no variables significantly associated with fertility consultation on univariate analysis. With only n=17 fertility consults in this group, not all variables could be included in the model due to low event numbers and multivariable analysis was not indicated.

Supplemental Table 5. Variables associated with live birth among female AYA

|  | Univariate | | Multivariable | |
| --- | --- | --- | --- | --- |
|  | HR (95%CI) | p-value | HR (95%CI) | p-value |
| Age at diagnosis (per year) | 1.1 (1.0-1.2) | 0.07 | 1.1 (1.0-1.2) | 0.30 |
| Time period at diagnosis |  |  |  |  |
| Early (1992-1998) | ref | ref | Ref | ref |
| Middle (1999-2005) | 0.8 (0.6-1.2) | 0.33 | - | - |
| Late (2006-2011) | 0.8 (0.5-1.2) | 0.31 | - | - |
| Locus of Care |  |  |  |  |
| Pediatric | 0.7 (0.4-1.0) | 0.06 | 0.8 (0.5-1.3) | 0.33 |
| Regional Cancer Center | ref | ref | Ref | ref |
| Community Center | 0.9 (0.6-1.3) | 0.53 | 0.9 (0.6-1.2) | 0.43 |
| Neighborhood income quintile |  |  |  |  |
| Rural | 1.3 (0.8-2.2) | 0.36 | 1.2 (0.7-2.1) | 0.51 |
| Urban Q1 (lowest) | 1.4 (0.8-2.4) | 0.23 | 1.4 (0.8-2.4) | 0.22 |
| Urban Q2 | 0.9 (0.5-1.4) | 0.56 | 0.8 (0.5-1.4) | 0.42 |
| Urban Q3 | 1.5 (0.9-2.3) | 0.09 | 1.4 (0.9-2.3) | 0.12 |
| Urban Q4 | 1.0 (0.6-1.5) | 0.90 | 0.9 (0.6-1.4) | 0.79 |
| Urban Q5 (highest) | ref | ref | Ref | ref |
| Region |  |  |  |  |
| Central | ref | ref | Ref | ref |
| East | 1.1 (0.7-1.7) | 0.76 | - | - |
| North | 1.2 (0.7-2.1) | 0.54 | - | - |
| Toronto | 0.8 (0.4-1.5) | 0.54 | - | - |
| West | 1.3 (0.9-1.9) | 0.14 | - | - |
| B symptoms |  |  |  |  |
| No | ref | ref | Ref | ref |
| Yes | 1.1 (0.8-1.5) | 0.64 | - | - |
| Disease extent |  |  |  |  |
| Limited | ref | ref | Ref | ref |
| Advanced | 1.1 (0.8-1.5) | 0.38 | - | - |
| Maximum deposit size |  |  |  |  |
| ≤5 centimeters | ref | Ref | Ref | ref |
| 6-9 centimeters | 1.0 (0.6-1.6) | 0.99 | - | - |
| >10 centimeters | 1.3 (0.8-2.0) | 0.30 | - | - |
| Treatment modality |  |  |  |  |
| Chemotherapy | 0.9 (0.7-1.2) | 0.48 | - | - |
| Combined modality | ref | ref | Ref | ref |
| Seen by AYA expert |  |  |  |  |
| No | ref | ref | Ref | ref |
| Yes | 0.9 (0.5-1.4) | 0.55 | 1.0 (0.6-1.6) | 0.91 |

AYA – Adolescents and young adults; CI – confidence interval; HR – Hazard ratio
